# Multiple Mechanisms Driving F-actin-Dependent Transport of Organelles to and From Secretory Sites in Bovine Chromaffin Cells

**DOI:** 10.3389/fncel.2018.00344

**Published:** 2018-10-09

**Authors:** Yolanda Gimenez-Molina, José Villanueva, Maria del Mar Francés, Salvador Viniegra, Luis M. Gutiérrez

**Affiliations:** Instituto de Neurociencias, Centro Mixto CSIC-Universidad Miguel Hernández, San Juan de Alicante, Spain

**Keywords:** chromaffin cells, F-actin dynamics, organelle transport, vesicles, mitochondria, secretion

## Abstract

Neuroendocrine chromaffin cells represent an excellent model to study the molecular mechanisms associated with the exo-endocytotic cycle of neurotransmitter release. In this study, EGFP-Lifeact and confocal microscopy has been used to analyze the re-organization of the cortical F-actin cytoskeleton associated to organelle transport during secretion with unprecedented detail. In these cells secretory events accumulate in temperature-sensitive and myosin II-dependent F-actin expansions and retractions affecting specific regions of the sub-membrane space. Interestingly, not only vesicles but also mitochondria are transported toward the plasmalemma during these expansions. Simultaneously, we found F-actin cytoskeletal retraction withdraws vesicles from the sub-plasmalemmal space, forming novel empty internal spaces into which organelles can be transported. In addition to these well-coordinated, F-actin-myosin II dependent processes that drive the transport of the majority of vesicles, fast transport of chromaffin vesicles was observed, albeit less frequently, which used F-actin comet tails nucleated from the granular membrane. Thus, upon cell stimulation F-actin structures use diverse mechanisms to transport organelles to and from the membrane during the exo-endocytotic cycle taking place in specific areas of cell periphery.

## Introduction

The cytoskeleton plays a key function in exocytosis and the molecular events that control it. In neuroendocrine cells, F-actin was initially considered to fulfill a role as a cortical barrier that prevents the access of secretory vesicles (SVs) to actives sites (Trifaro et al., 1985; Cheek and Burgoyne, 1986; Aunis and Bader, 1988). This barrier was shown to be partially disassembled by altering the activity of F-actin cross-linkers, such as fodrin (Perrin and Aunis, 1985) or/and by enhancing the activity of F-actin severing proteins (Vitale et al., 1991; Zhang et al., 1996), and results in the access of vesicles to form part of the ready-releasable pool (Vitale et al., 1995). This vision has evolved as the cytoskeleton has become considered as a more dynamic element and it has been attributed a role in transport based on the interaction of actin fibers with SVs (Steyer and Almers, 1999; Lang et al., 2000; Oheim and Stuhmer, 2000; Johns et al., 2001). To act as both a retentive barrier and a transport system, cortical F-actin must undergo a profound re-organization during the secretory cycle. This involves the shift from a dense cortical structure parallel to the plasma membrane in the resting state to a more open structure with spaces that allow vesicles to cross it and reach the membrane when the cell is stimulated (Giner et al., 2005). These transitory changes in the disposition of F-actin are governed by the motor activity of myosin II molecules, which is mediated by the phosphorylation of the regulatory light chain in a calcium-dependent manner (Cote et al., 1986; Gutierrez et al., 1989). In addition, other molecular motors like myosin V are essential to transport granules from the cell interior to the periphery in chromaffin cells in order to replenish the vesicle pools exhausted by secretion (Rose et al., 2003; Rudolf et al., 2003).

In addition to the possible transport of chromaffin vesicles along actin tracks or fibers, F-actin could also transport vesicles that appear to be trapped inside cages formed in the peripheral cytoskeleton (Giner et al., 2007), thereby following the dynamics of the overall F-actin structure. Similarly, other studies support the idea that entire regions of the cytosol could transport vesicles as if on a conveyor belt, therefore vectoring the overall transport of vesicles during exocytosis toward the cell periphery (Maucort et al., 2014). More recently, a new potential transport mechanism was described based on the coordinated displacement of F-actin structures embedding chromaffin granules, a process defined as a “cast” system (Papadopulos et al., 2015). Therefore, the transport of vesicles by F-actin in neuroendocrine cells appears to occur via distinct mechanisms, probably as a function of the different regions of the cytosol in which it is acting.

The goal of this study was to reveal the different F-actin structures and mechanisms that cooperate in the transport of chromaffin granules during the secretory process taking place in specific areas of the cell periphery. Our results highlight the concept that multiple mechanisms cooperate to transport not only chromaffin vesicles but also other organelles, such as mitochondria, which appear to be important in shaping the dynamics of the secretory process in active zones of the cell membrane.

## Materials and methods

### Chromaffin cell transfection and culture

Chromaffin cells were isolated from bovine adrenal glands by collagenase digestion and they were separated from the debris by centrifugation on Percoll gradients, as described previously (Gutierrez et al., 1995, 1998; Gil et al., 1998, 2002). The cells were maintained as monolayer cultures in Dulbecco's modified Eagle's medium (DMEM) supplemented with 10% fetal calf serum (FCS), 10 μM cytosine arabinoside, 10 μM 5-fluoro-29-deoxyuridine, 50 U/mL penicillin, and 50 mg/mL streptomycin. The cells were harvested and plated at a density of 150,000 cells/cm^2^ on 22 mm diameter poly-lysine coated coverslips, and they were studied between the third and sixth day after plating.

To perform the experiments, the cell culture medium was replaced with Krebs/HEPES (K/H) basal solution containing (in mM): NaCl, 134; KCl, 4.7; KH_2_PO_4_, 1.2; MgCl_2_, 1.5; CaCl_2_, 2.5; glucose, 11; ascorbic acid, 0.56; and Na-HEPES, 15 [pH 7.4]. The cells were stimulated by depolarization using a K/H solution with high K^+^ (59 mM) obtained by iso-osmotically replacing NaCl by KCl in K/H basal solution. Stimulation protocol was applied between 10 and 50 s in the 60 s total time shown in the recordings.

### Dynamic confocal imaging of the F-actin cytoskeleton, vesicles, and mitochondria

F-actin structures were labeled with the EGFP-tagged, 17 amino acid LifeAct peptide that binds to F-actin without altering its dynamics *in vivo* or *in vitro*, as described previously (Riedl et al., 2008). Dense-core vesicles were labeled by NPY–mRFP plasmid transfection, as described elsewhere (Taraska et al., 2003; Aoki et al., 2010). Myosin II activity and location were studied by expression of a tagged fluorescent form of the wild type regulatory subunit of myosin II (pRLC-wt-DsRed), and a mutant non-active unphosphorylatable form (pRLC-T18A-S19A-DsRed) (Neco et al., 2004) in double expression experiments with EGFP-LifeAct. Single and dual transfection of isolated chromaffin cells using the Amaxa basic nucleofector kit (Lonza, red VVPG-1001) served to visualize the F-actin cytoskeleton and chromaffin granules in living primary mammalian neurons (Program 0–005, Amaxa GmbH. Koehl, Germany).

Two days after transfection, the cells were incubated for 15 min with Mitotracker Red FM (0.1 μM: Invitrogen-Molecular Probes ref M22425), to visualize the mitochondria in live cells as described previously (Villanueva et al., 2014). Fluorescence confocal images were obtained with an Olympus Fluoview FV300 confocal laser system mounted on an IX-71 inverted microscope incorporating a 100X PLAN-Apochromatic oil-immersion objective with 1.45 NA. Excitation was achieved with argon and helium-neon visible light lasers, and dual fluorescence was registered by sequential acquisition using a 488 nm argon ion 40 mW to excite EGFP and a 543 nm He/Ne 10 mW for RFP.

Images were recorded from live cells at two temperatures (22 and 30°C) to evaluate the effect of temperature on F-actin dynamics. The pharmacological agents used were jasplakinolide (1 μM for 15 min: Calbiochem, ref 420107), blebbistatin (5 μM for 30 min: Calbiochem ref 203391), and paclitaxel (taxol, 1 μM for 30 min: Sigma ref T-7402), minimal concentrations and incubations to reach maximal specific effect.

In some experiments, cells where stimulated at different periods and the secretory process stopped by addition of ice-cooled K/H buffer lacking CaCl_2_. Then, cells where incubated during 2 h with anti-Dopamine β-Hydroxylase (DβH) rabbit polyclonal antibodies (1:500 dilution in the same ice cooled buffer, Millipore AB 1585, Lot 2826440). Cell were fixed using 4% paraformaldehyde PBS solution before a secondary antibody coupled to AlexaFluor 546 at a 1:200 dilution (InvitroGen ref A11010) was incubated during 1.5 h at room temperature to visualize DβH labeling in confocal microscopy.

### Data analysis from confocal microscopy images

All microscopy images were analyzed with Image J software (Collins, 2007) using integrated measurements (particle analyzer, perimeter, area, mean gray value, and threshold), and some plug-ins (multitracker, colocalization analysis, and 3D reconstruction) to: automatically detect and quantify particles and cavities in thresholded images; calculate the area and perimeter; and measure the variations in distance and intensity (software downloaded from. https://imagej.nih.gov/ij/). Finally, we also used the Confocal Uniovi Image J version for 2D projections and 3D reconstructions of the F-actin structural responses linked to local F-actin dynamics. Displacements of F-actin fibbers, vesicles and cortical mitochondria populations linked to cortical F-actin dynamics under stimulation are shown as distance variations with our scale of 26 pixels/μm. Firstly, we measured distances between an internal static point near the nucleus called “reference” to the: F-actin external profile line, individual vesicle or mitochondria centroid included at cortical active zones for expansion and retraction events. These distances were measured at 5, 10, 20, 30, 40, 50, 55 and 60 s. Then distance variations were obtained by subtracting each one to the own distance at 10 s (stimulus start time).

Graphs were obtained with Graphpad Prism (GraphPad software, San Diego, CA, USA) and Adobe Photoshop 7.0. A two way ANOVA test was used to establish the significance of the experimental data (considered significant at *p* < 0.05). Significance level symbol meaning: n.s: *P* > 0.05, ^*^P ≤ 0.05, ^**^P ≤ 0.01, ^***^P ≤ 0.001, ^****^P ≤ 0.0001. The data were expressed as the mean ± SEM values from experiments performed on individual cells (n cells), and on single expansion and retraction events (n events), from at least three different cultures.

### Ethics statement

Adrenal glands were obtained from an industrial slaughterhouse (Matadero de Orihuela SA) subjected to strict regulations of the Ministries of Agriculture, Industry and Health of Spain in accordance with EC normative.

All the protocols described in this article were approved by the Organo Evaluador de Proyecto of University Miguel Hernández, the office in charge of the observation of the Ethics in animal care and experimentation in our institution.

## Results

### Chromaffin cell stimulation evokes local changes in the F-actin cortical cytoskeleton without affecting its overall profile

**Figure 1:**
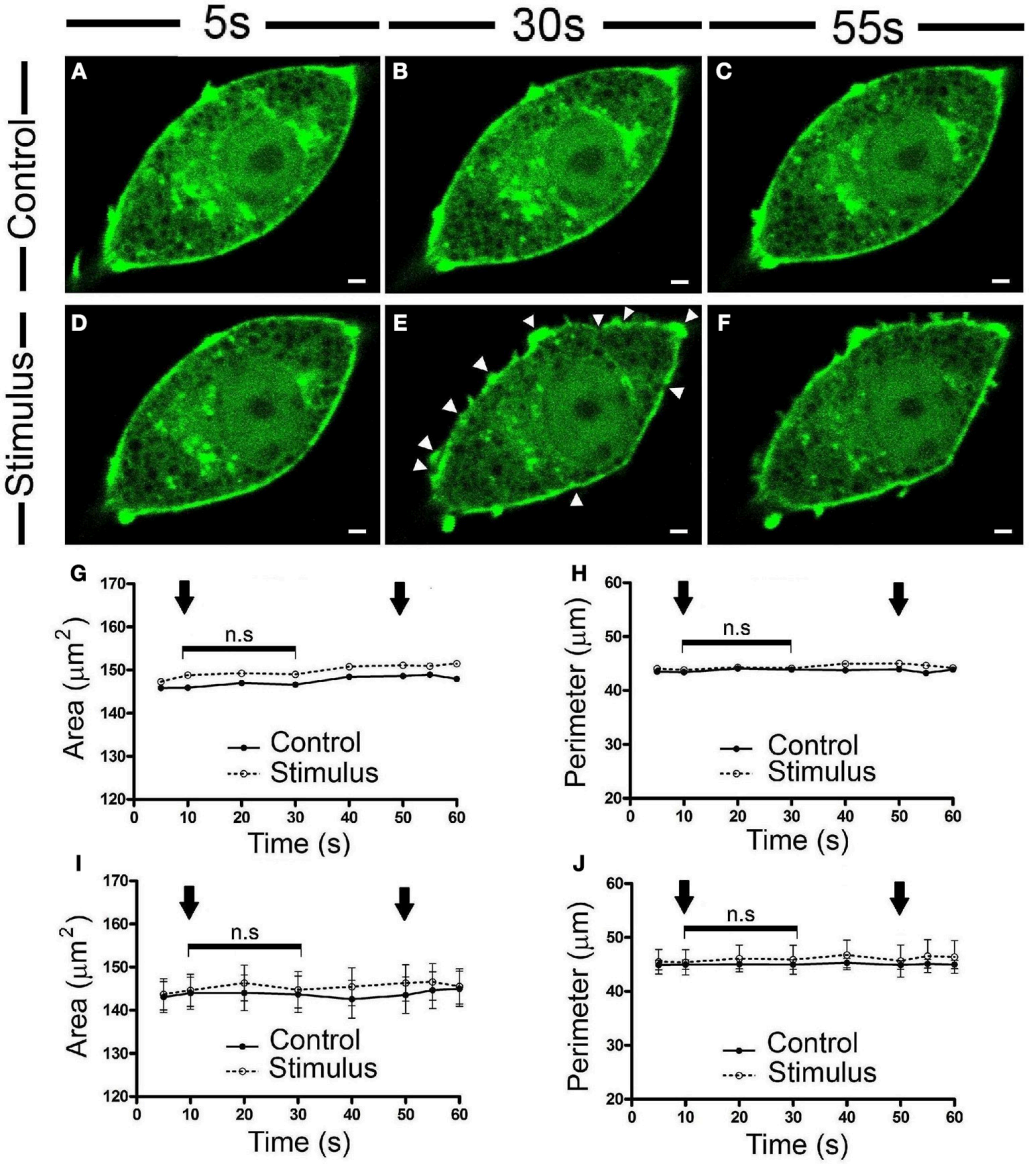
Chromaffin cell stimulation promotes local changes in the cortical F-actin cytoskeleton without varying the overall cell profile. **(A–F)** Confocal fluorescence images of a control **(A–C)** and stimulated chromaffin cell by KCl depolarization **(D–F)** expressing EGFP-LifeAct to show F-actin (green) at temporal series of 5 s **(A,D)**, 30 s **(B,E)**, and 55 s **(C,F)** for a 60 s total time recordings at room temperature (22°C). White triangles show local changes on F-actin cortical profile. **(G–H)** Changes in the area **(G)** and perimeter **(H)** for this representative cell, under control and stimulation conditions. Arrows indicate the beginning and the end of stimulation. **(I–J)** Mean ± SEM values of the area **(I)** and perimeter **(J)** for control (*n* = 20 cells) and stimulated cells (*n* = 20 cells). Statistical significance was assessed by Two way ANOVA: n.s, non-significant results. Scale bars represent 1 μm.

To carry out a detailed study of the subtle changes to the F-actin chromaffin cytoskeleton provoked by cell stimulation, we assessed the EGFP-LifeAct expression in cultured bovine chromaffin cells by fluorescence confocal microscopy after depolarizing the cells with KCl at room temperature (22°C), since this is the most common experimental condition used in bibliography. Rapid inspection of the images revealed no obvious changes in the F-actin labeling of control unstimulated cells (Figure 1A), whereas changes to the peripheral F-actin cortex were evident in stimulated cells (Figure 1B, Movie 1), not apparently associated with an increase in cell size. The absence of a change in overall size was further evidenced when the temporal evolution of the area and perimeter of the cells was analyzed (Figures 1G,H), the averaged values corresponding to 20 cells from 3 different cultures (Figures 1I,J).

**Figure 2:**
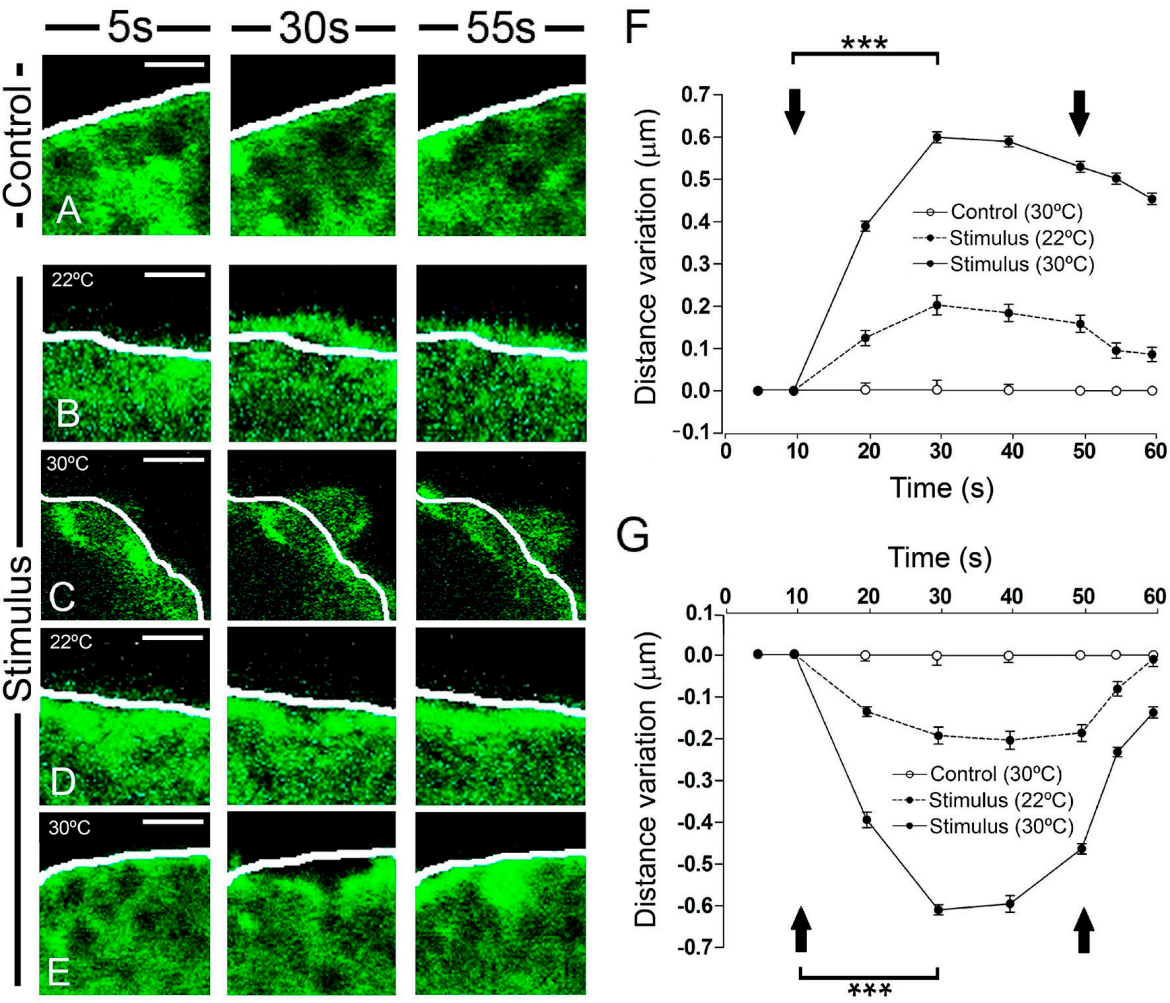
Time course and temperature dependence of the local changes at the F-actin cortical barrier. **(A–E)** Confocal fluorescence images acquired in the equatorial plane of cultured chromaffin cells expressing EGFP-LifeAct to show F-actin cortical barrier (green) from a control cell **(A)** and stimulated cells by KCl depolarization **(B–E)** for 5 s, pre-stimulus; 30 s, stimulus; and 55 s, post-stimulus. **(B–E)** Expansion **(B–C)** and retraction **(D–E)** events developed in stimulated cells were analyzed individually for 22°C **(B**, expansion; **D**, retraction) and 30°C **(C**, expansion; **E**, retraction) to study the temperature influence. White lines indicate the initial position (t_0_) of F-actin cortical barrier profile for each register. F-actin displacements magnitude was measured in terms of distance variation at active zones of expansions and retractions. **(F,G)** Mean ± SEM of the normalized distance variation of the F-actin cortical barrier for expansion **(F)** and retraction **(G)** events in control (*n* = 15 cells, *n* = 30 sections) and stimulated cells for 22°C (*n* = 15 cells, *n* = 48 expansions, *n* = 50 retractions) and 30°C (*n* = 15 cells, *n* = 60 expansions, *n* = 57 retractions). Arrows indicate the beginning and end of stimulation. Statistical significance was assessed by Two way ANOVA: ^***^P < 0.001. Scale bars: 1 μm.

A more detailed inspection of the peripheral F-actin structure revealed clear local changes in terms of expansions or retractions (Figure 2). The most external aspect of the peripheral F-actin profile extended in particular zones, extending about 0.2 μm from its initial position when stimulated at room temperature (22°C: Figures 2B,F, as indicated by the white line). No such change was seen in control unstimulated cells. By contrast, in other areas of the stimulated cells there was a retraction of the cortical F-actin band of a similar magnitude relative to the initial reference position indicated by the white line (Figures 2D,G). Both these changes were transient and they reached a maximum around 20 s after initiating the stimulus, with partially recovery toward the resting level within tenths of a second. These effects were enhanced when the temperature was increased to 30°C, in which case displacements of around 0.6 μm were evident (representing a speed of 0.03 μm/s). This represented a 3-fold enhancement in F-actin displacement when compared to the expansion and retraction at room temperature (see Movie 2). As mentioned earlier for 22°C experiments, changing the temperature to 30°C did not affect significantly the magnitude of the overall cellular profile and area (data not shown).

### Secretory events accumulate in the membrane associated with cortical expansions and retractions

Are these expansions and retractions specifically associated with areas of secretory activity?. This is a relevant question since changes in the cell periphery shape has been reported in a multitude of cell types and could be completely unrelated with the secretory function. Therefore, we conceived experiments in the conditions reported above and including the detection of secretory activity by using inmunolocalization of the vesicular membrane incorporated into the plasmalemma after exocytosis using antibodies again dopamine β-hydroxylase (DβH) as described elsewhere (Villanueva et al., 2014).

**Figure 3:**
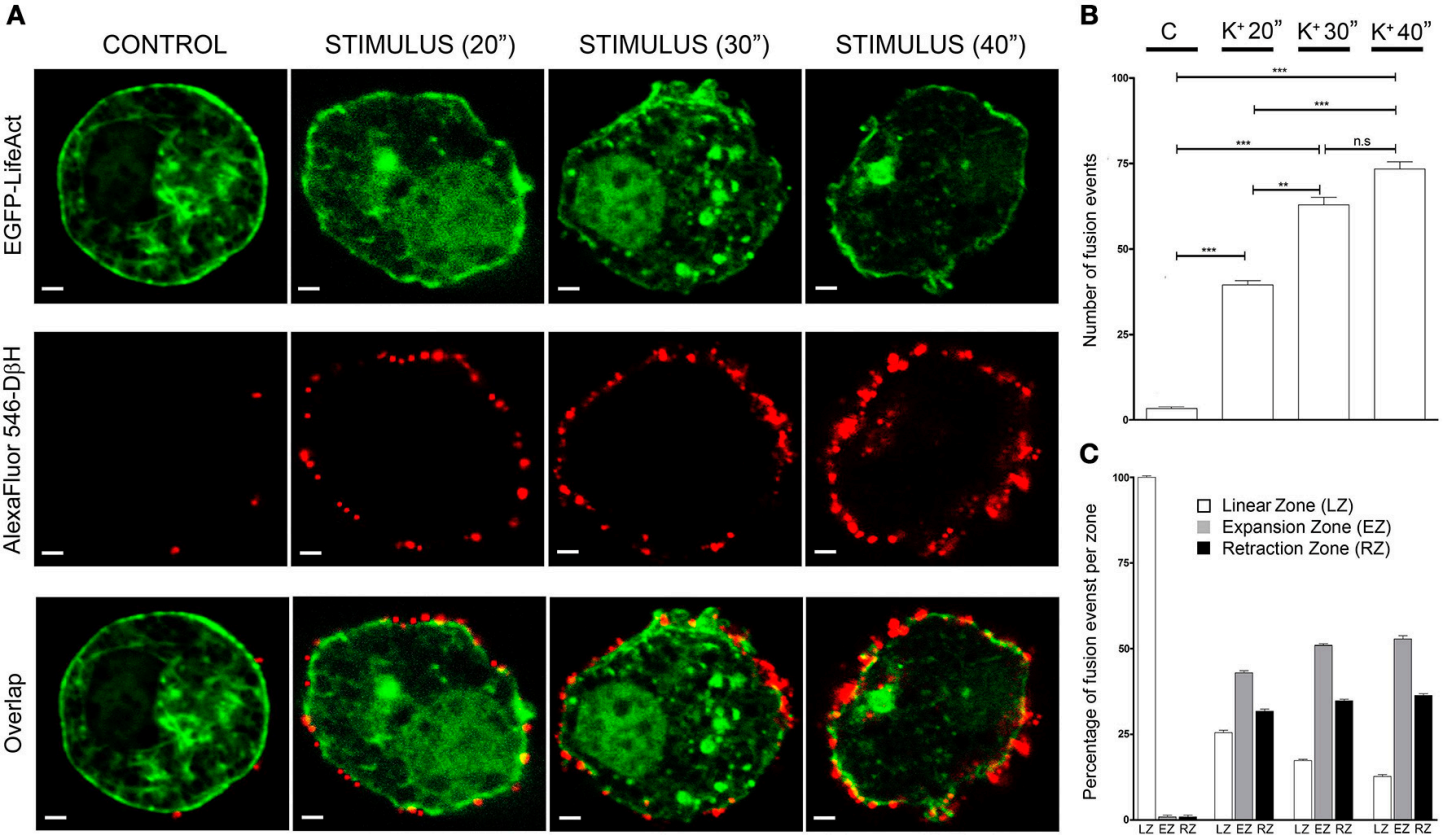
Expansion and retraction areas are active zones for exocytosis**. (A)**, Confocal fluorescence images of the F-actin cytoskeleton (green) and Dopamine β-Hydroxylase (DβH) incorporated to the plasma membrane after exocytosis (red) from fixed cultured chromaffin cells expressing EGFP-LifeAct and then immunolabeled with anti-DβH (secondary antibodiy coupled to AlexaFluor 546) for control and KCl stimulation at different periods (20, 30, and 40 s). **(B)**, Mean ± SEM values of the total number of fusion events measured in every condition [*N* = 10 control cells; *N* = 10 stimulated cells (20 s); *N* = 10 stimulated cells (30 s); *N* = 10 stimulated cells (40 s)]. **(C)**, Mean ± SEM values of the percentage of fusion events studied in linear zones(LZ), expansion zones (EZ), and retraction zones (RZ) under each experimental condition. Statistical significance was assessed by Two way ANOVA; n.s: *P* > 0.05, ^**^P ≤ 0.01, ^***^P ≤ 0.001.

Cells incubated with non-depolarizing media during 30 s periods presented very few secretory events as detected by anti-DβH staining in equatorial images (red dots in Figure 3A CONTROL), and during cell stimulation by depolarization with a 59 mM KCl solution for increasing time periods the secretory events multiply by 10 times at the shorter period (20 s) of stimulation and even more during 40 s stimulations (20 times, Figure 3B). Interestingly, stimulated cells showed expansions and retractions areas as indicated in the previous figures and therefore we performed an analysis of the frequency of secretory events localizing in areas with the presence of expansions and retractions in comparison with their presence in the peripheral areas lacking these alterations in the cell periphery. Control unstimulated cells present a regular circular profile and therefore the infrequent secretory events were largely present in areas devoid of expansions and retractions (Linear zones, Figure 3C). Upon stimulation, secretory events tend to take place in both expansions and retractions zones (EZ and RZ in Figure 3C) and these tendency increases at prolonged stimulations periods. Therefore in cultured chromaffin cells, the secretory activity seems to present a preferential localization in association with the F-actin expansions and retractions areas present in the cell cortex.

### Myosin II appears to control the expansion and retraction of F-actin

**Figure 4:**
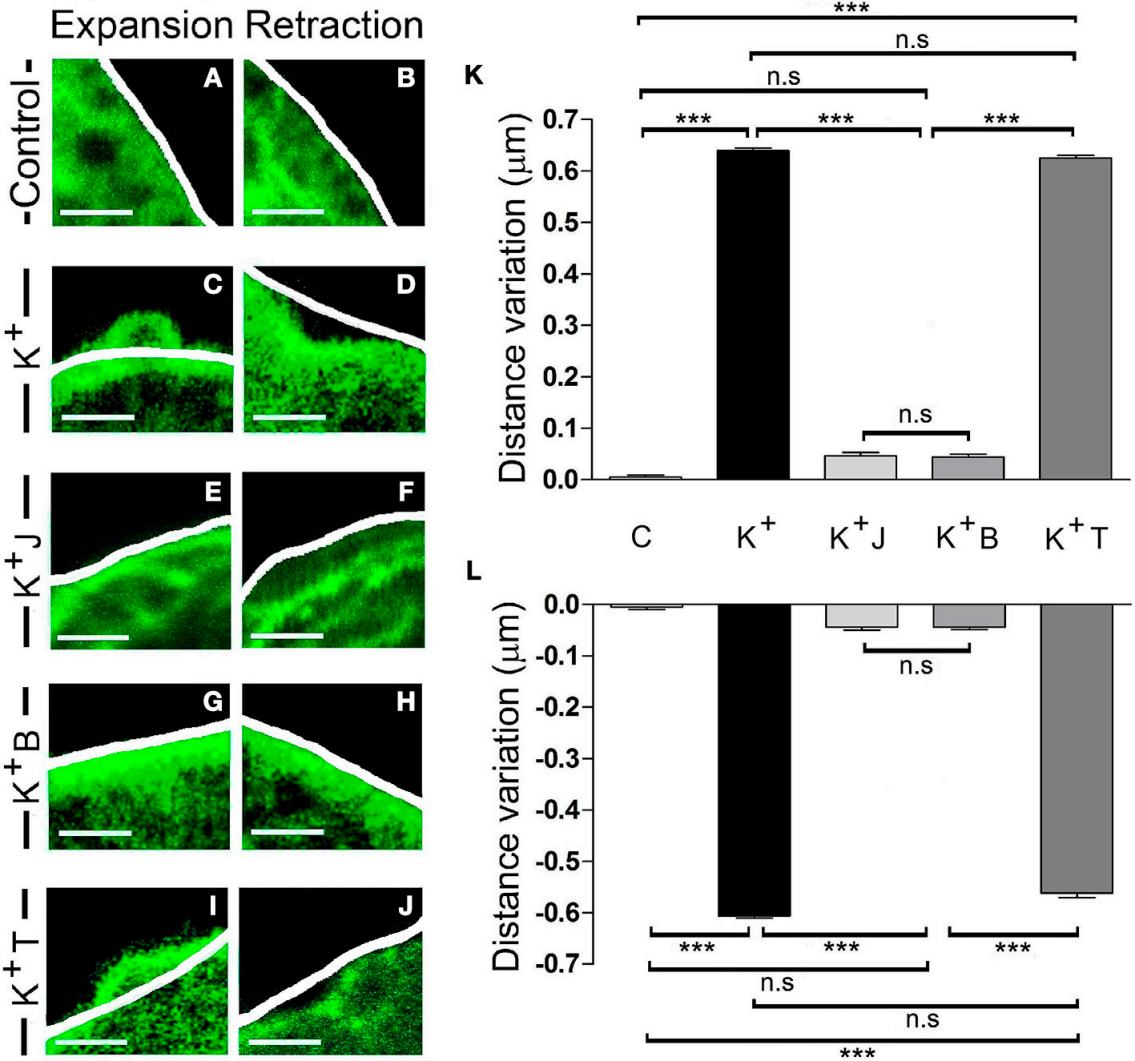
F-actin and myosin II activity controls expansion and retraction. **(A–J)** Confocal fluorescence images of the F-actin cortical barrier from cultured chromaffin cells expressing EGFP-LifeAct in control **(A,B)** and stimulated cells by KCl depolarization **(C–J)** at maximum development time (30 s) for both expansion **(A,C,E,G, and I)** and retraction **(B,D,F,H,J)** events registered at 30°C. **(C–J)** Stimulated cells group includes cells without any pharmacological treatment **(C–D)** and others previously treated with jasplakinolide 1 μM for 15 min **(E–F)**, blebbistatin 5 μM for 30 min **(G–H)** and taxol 0.1 μM for 30 min **(I–J)**. White lines indicate the initial position (t_0_) of F-actin cortical barrier profile for each register. F-actin displacements magnitude for expansion and retraction events were measured in terms of the distance variation value at 30 s linked to F-actin dynamics under each established condition.**(K–L)** Mean ± SEM of the normalized distance variation value of the F-actin cortical barrier at 30s for expansions **(K)** and retractions **(L)** in control cells (*n* = 10 cells, *n* = 40 sections), untreated stimulated cells (*n* = 10 cells, *n* = 50 expansions, *n* = 46 retractions) and stimulated cells previously treated with jasplakinolide (*n* = 10 cells, *n* = 40 zones), blebbistatin (*n* = 10 cells; *n* = 40 zones), and taxol (*n* = 10 cells, *n* = 45 expansions, *n* = 40 retractions). Statistical significance was assessed by Two way ANOVA: ^***^P < 0.001; n.s: non-significant results. Scale bars: 1 μm.

In order to test if these local changes are associated with the activity of the F-actin cytoskeleton, we evaluated the effect of chemicals that specifically interact with F-actin or its molecular motor, myosin II. We first incubated cells expressing EGFP-LifeAct for 15 min with the F-actin stabilizer jasplakinolide (1 μM) at room temperature (Bubb et al., 1994; Zhang et al., 2017). When the F-actin dynamics were assessed by confocal microscopy, the changes measured in expansions (Figure 4K) and retractions (Figure 4L) that were induced by stimulation (Figures 4C,D), and are absent in control non-stimulated cells (Figures 4A,B), were almost totally abolished in the presence of this compound, as shown by the variations in distance relative to the initial profile (see white line in Figures 4E,F). Hence, it would appear that F-actin activity drives these changes. Moreover, in cells exposed to blebbistatin (5 μM) for 30 min, a specific inhibitor of myosin II (Limouze et al., 2004; Villanueva et al., 2012) the F-actin displacements were again impaired (Figures 4G,H). Conversely, in the presence of the microtubule stabilizer paclitaxel (0.1 μM), 30 min: Horwitz et al., 1982; Neco et al., 2003, no changes were observed in the extent of expansion or retraction in response to the depolarization of these cells (Figures 4I,J). These results indicate that the local expansions and retractions in cortical F-actin were provoked by the dual activity of F-actin and myosin II, whereas other major cytoskeletal elements like microtubules were not involved.

In order to further confirm the key role of myosin II controlling F-actin dynamics during the formation of expansions and retractions we over-expressed in our cells a fluorescence tagged form of the wild-type regulatory subunit of myosin II (pRLC-wt-DsRed), and a mutant unphosphorylatable form (pRLC-T18A-S19A-DsRed) (Neco et al., 2004). The expression of this mutated form has a dramatic influence in F-actin dynamics (see Movie 4) and in our experiments completely abolished the local expansions and retractions while the expression of the fluorescence tagged wild type was fully active supporting these events (Supplementary Figure S1 and Movie 3). In consequence, it is clear that the dynamics of the binome F-actin-myosin II are essential for the molecular mechanism that govern local expansions and retractions.

### The generation of new empty spaces in conjunction with local changes in cortical F-actin

**Figure 5:**
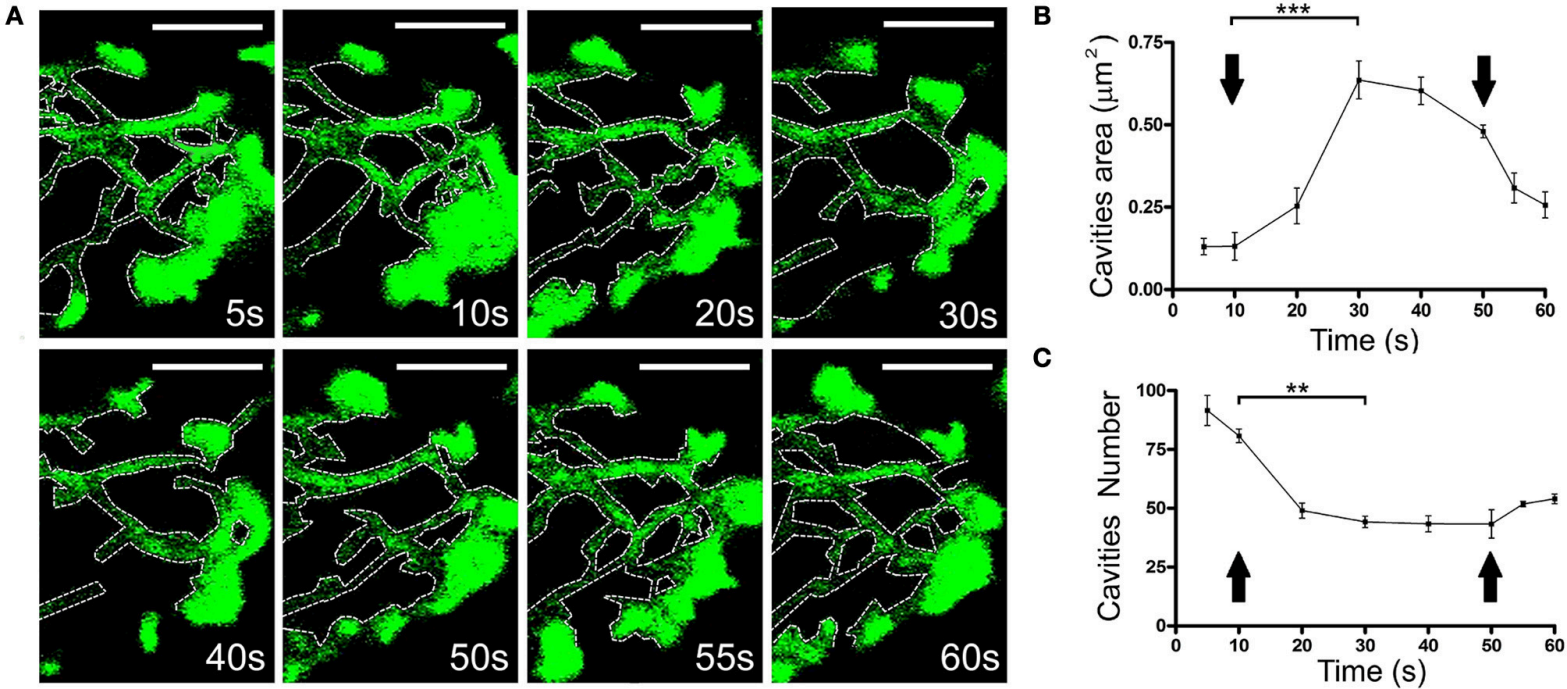
Change in the size and number of the F-actin cytoskeletal cavities during stimulation. **(A)** Time-lapse confocal fluorescence images of a cortical section in the equatorial plane from a chromaffin cell expressing EGFP-LifeAct under stimulation conditions by KCl depolarization at 30°C. Cytoskeleton cavities was detected by threshold and analyzed in terms of area and number at: 5, 10, 20, 30, 40, 50, 55, and 60 s. White discontinuous lines show cavities profiles inside the F-actin network. **(B,C)** Mean ± SEM values of area **(B)** and number **(C)** of cavities in stimulated cells (*n* = 10 cells, 35 zones, 306 cavities). Arrows indicate the beginning and end of stimulation. Statistical significance was assessed by Two way ANOVA:^**^P < 0.01, ^***^P < 0.001. Scale bars: represent 1 μm.

It would be expected that the displacement of F-actin structures has an impact on the presence of F-actin structures in the adjacent regions. Thus, we assessed the possible re-organization of F-actin in cells expressing EGFP-LifeAct. The changes that affect F-actin disposition followed similar dynamics to those reported for local changes in earlier studies (Figure 5A and Movie 5). These changes were related to the increase in the area of the pre-existing cavities (Figure 5B) and the decrease in their number (Figure 5C). Similarly, we detected the infrequent formation of new cavities that appeared to be generated from sub-plasmalemmal F-actin retraction, and the appearance of rings (Supplementary Figure S2). Moreover, it was also evident that vesicles could be trapped inside these structures, moving accordingly and that they were linked to their F-actin borders (Movie 6).

### Local F-actin dynamics promotes a sub-plasmalemmal displacement of vesicles

**Figure 6:**
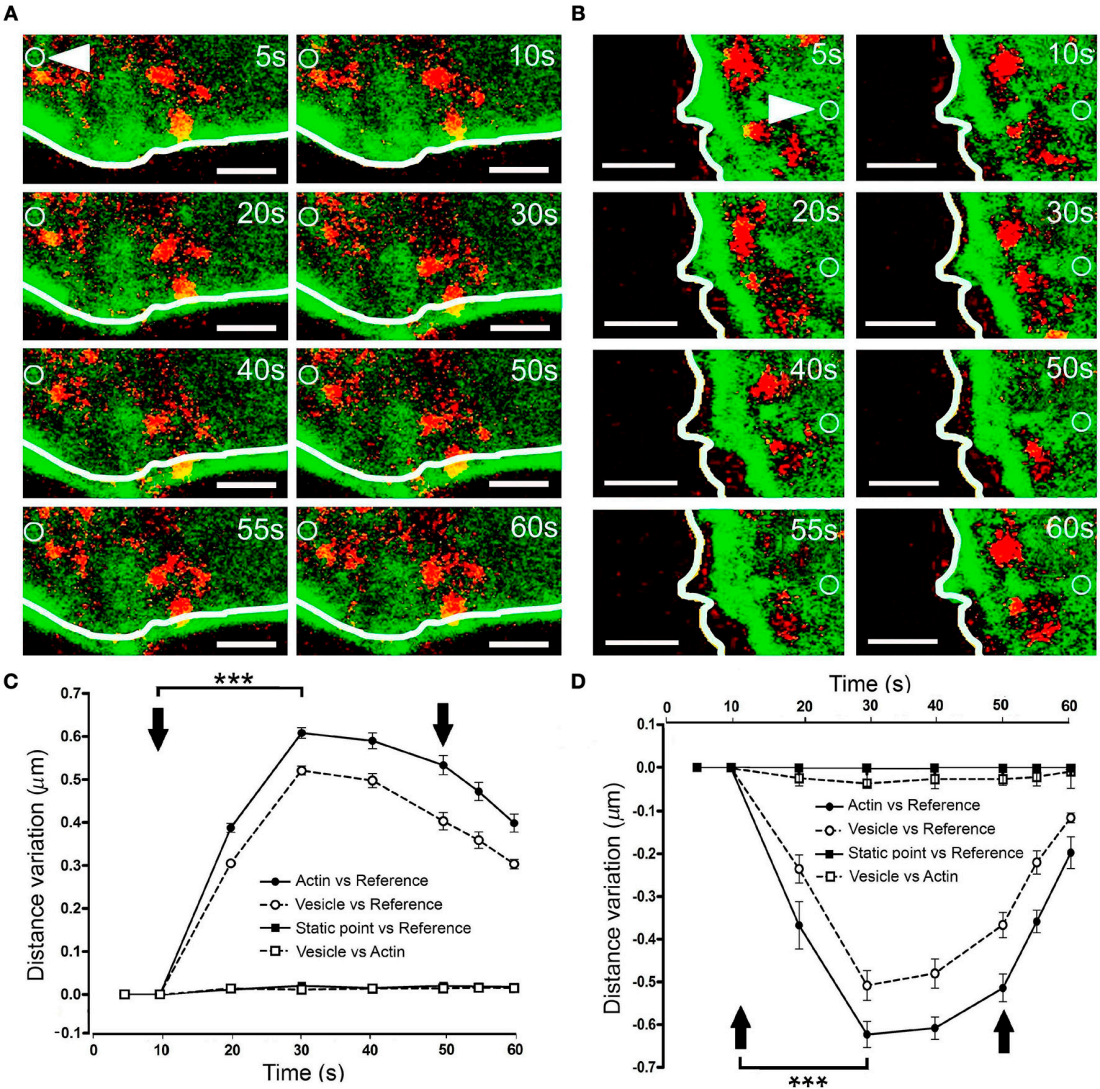
Vesicle displacement during cortical F-actin cytoskeleton retraction and expansion. **(A–B)** Time-lapse confocal fluorescence images of a representative expansion **(A)** and retraction **(B)** events from stimulated chromaffin cells expressing EGFP-LifeAct (green) and RFP-NPY (red) to show F-actin (green) and vesicles (red). Cells were stimulated by depolarizing with KCl at 30°C. White lines indicate the initial position (t_0_) of F-actin cortical barrier profile for each register and white arrows show an internal static zone as a control for cell movement. Local displacements of F-actin fibbers and cortical vesicles were measured individually in terms of distance variations to study movement magnitude for each one derived from expansion and retraction events and also between them in order to detect linked movements during F-actin dynamics. **(C,D)** Mean ± SEM values of the distance normalized variation for expansion **(C)** and retraction **(D)** events (*n* = 15 cells, *n* = 40 expansions, *n* = 37 retractions, *n* = 278 vesicles) Statistical significance was assessed by two way ANOVA: ^***^P < 0.001. Arrows indicate the beginning and end of stimulation. Scale bars: represent 1 μm.

To assess whether these F-actin displacements drive vesicles toward the plasma membrane, we co-expressed EGFP-LifeAct and RFP-NPY in our cells, thereby labeling F-actin and chromaffin granules. When expansion was obtained by KCl stimulation of a cell at 30°C, there was a clear displacement of the cortical F-actin zone around 0.6 μm from its initial position (as indicated by the white line describing the F-actin profile in Figure 6A, Movie 7). Interestingly, vesicles displayed a similar maximal displacement of 0.5 μm 20 s after applying the stimulus (Figure 6C). Furthermore, when measured relative to the position of the F-actin barrier at each moment, there was no detectable displacement between the two markers (Figure 6C, vesicles and F-actin profile). Similar conclusions were reached when we studied the displacement of the vesicles in relation to F-actin retraction (Figures 6B,D). Again, measuring the variation in distance between the F-actin profile and the vesicle's centroid indicated that the transient movement of chromaffin granules during stimulation occurred in association with the cortical cytoskeleton. Therefore, it appears that the local displacement of cortical actin filaments in chromaffin cells, reflected by the regions of expansion and retraction, guide the movement of vesicles upon stimulation.

### Mitochondria also move in association with local F-actin displacements

**Figure 7:**
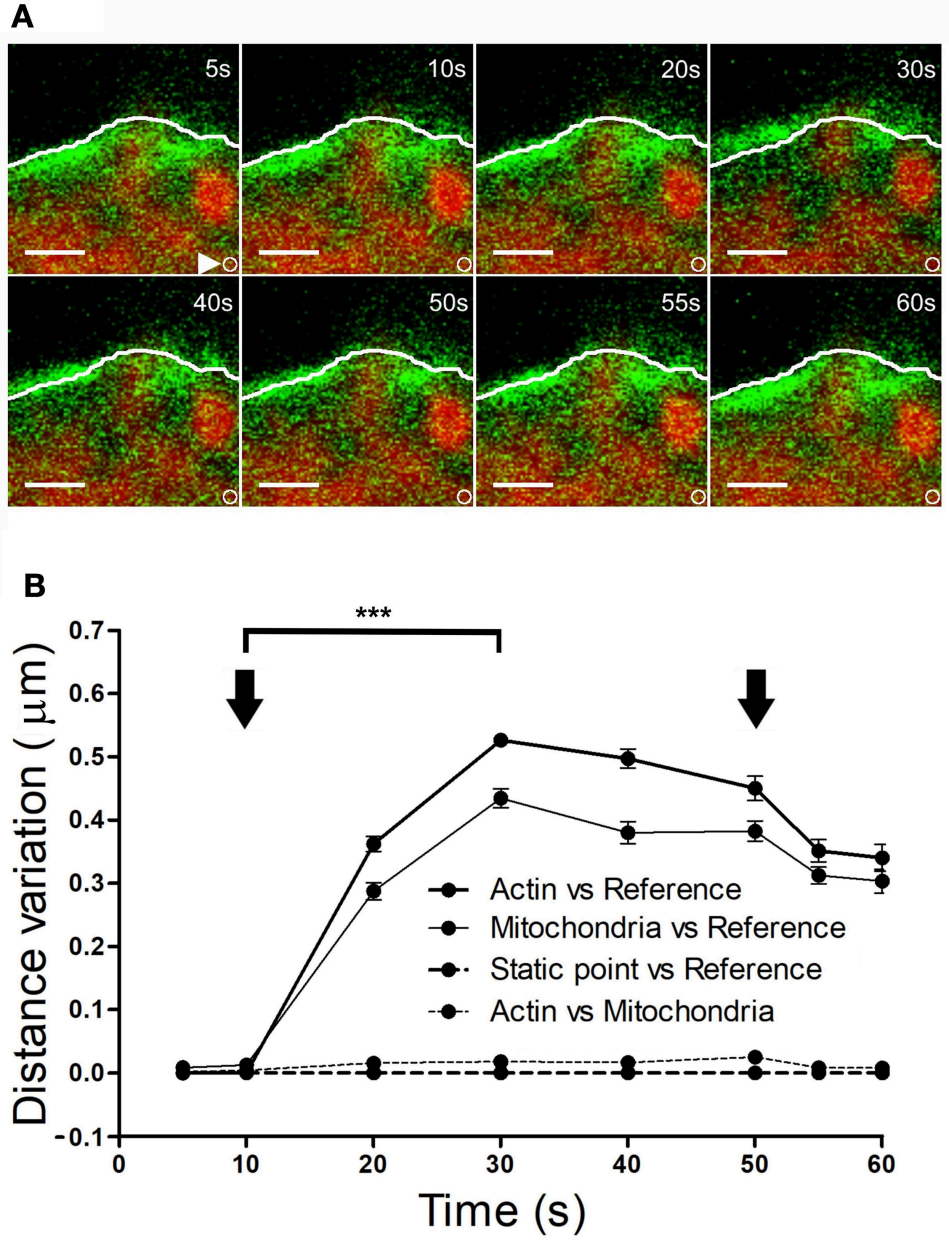
Mitochondrial displacement in association with the F-actin cortex. **(A)** Time-lapse confocal fluorescence images of a representative expansion zone acquired in an equatorial plane of a stimulated chromaffin cell expressing EGFP-LifeAct and then incubated with Mitotracker red to show F-actin (green) and mitochondria (red). The cell was stimulated by depolarizing with KCl at 30°C. White lines indicate the initial position (t_0_) of F-actin cortical barrier profile for each register and white arrows show an internal static zone as a control for cell movement. Local displacements of F-actin fibbers and cortical mitochondria populations were measured individually in terms of distance variations to study movement magnitude for each one and also between them in order to detect linked movements derived from expansions events. **(B)** Mean ± SEM values of distance normalized variation for expansion events in stimulated cells (*n* = 30 cells, *n* = 30 expansions, *n* = 34 mitochondrial populations). Arrows indicate the beginning and end of stimulation. Statistical significance was assessed by Two way ANOVA: ^***^P < 0.001. Scale bars: represent 1 μm.

It is conceivable that displacement of the F-actin cytoskeleton that carries chromaffin granules could also drive the movement of other organelles. To explore this possibility, we simultaneously labeled F-actin (as indicated above) and mitochondria using Mitotracker Red. KCl stimulation of cells at 30°C provoked displacement of the F-actin cortical cytoskeleton within 10–20 s (Figure 7A, at 10 s), which was estimated to move 0.4–0.45 μm relative to its initial position. Interestingly, the mitochondria observed in these images (in red) were also displaced to a similar extent, with an average mitochondrial displacement in cells from different cultures of c.a. 0.5 μm (Figure 7B). Again, the displaced mitochondria were associated to the cytoskeleton as they moved relative to static points, although the distance between these two elements remained constant. Interestingly, we did not observe mitochondrial movements associated with retractions, indicating the relative absence of mitochondria in the regions where cortical retraction occurred. Consequently, the changes in F-actin associated with local expansions drove the transport of organelles, implying the possible displacement of all cytosolic components in response to stimulation.

### Propelling tail comets, an exception to the conventional form of vesicle transport

**Figure 8:**
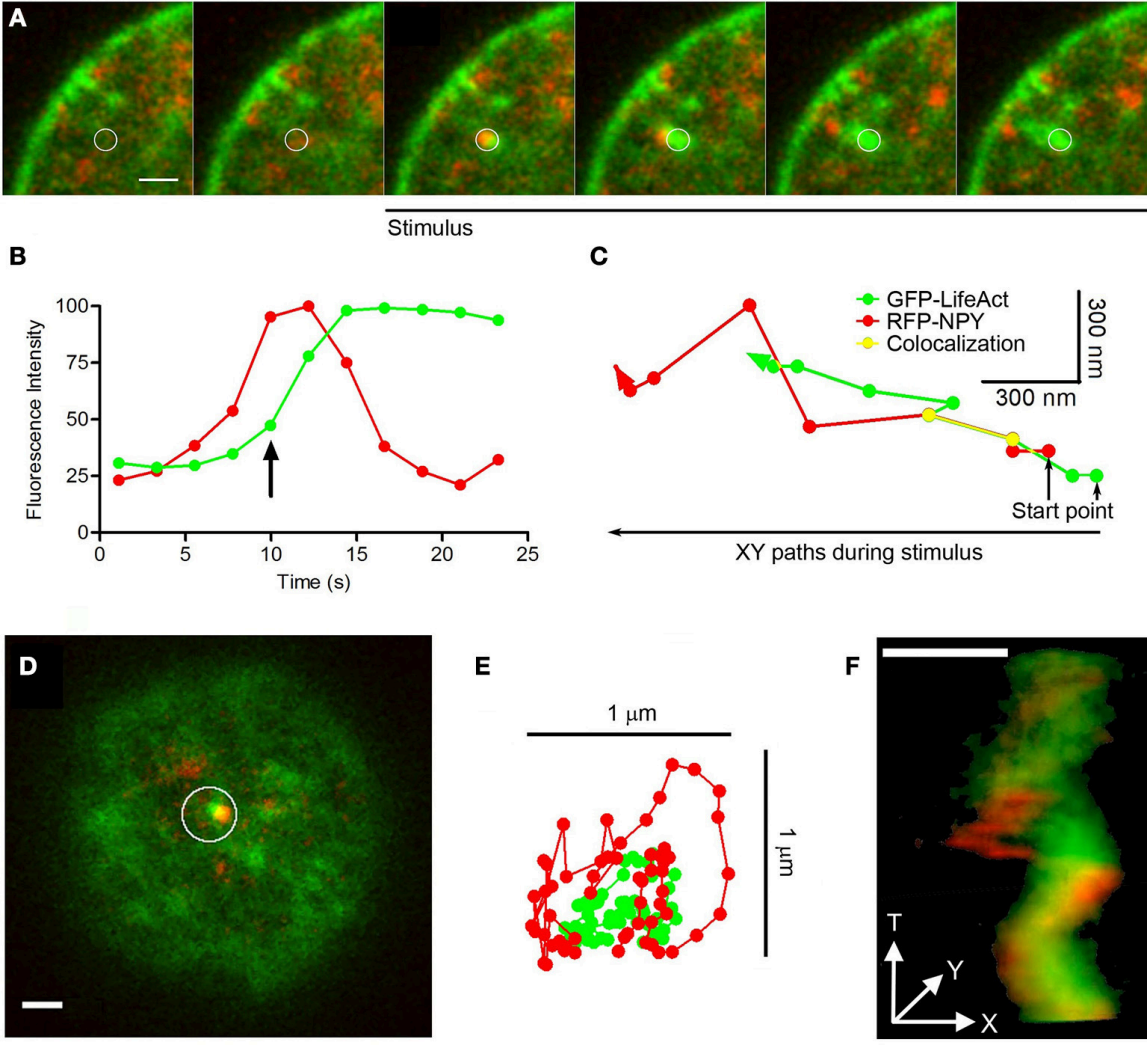
Comets: structure, mobility and vesicle linking. **(A)** Time-lapse confocal fluorescence images of a cortical section acquired in the equatorial plane from a stimulated chromaffin cell expressing EGFP-LifeAct (green) and RFP-NPY (red) to show F-actin (green) and vesicles (red). Cells were stimulated by depolarizing with KCl at 30°C. White circle indicates the region of interest (ROI) from this confocal images composition showing the vesicle arrival linked to a F-actin aggregate with a temporal development of a F-actin tail comet (green) propelling the vesicle (red) under stimulus. Images were acquired at 4 s intervals. **(B)** Temporal evolution of the red (vesicle) and green (F-actin) integrated fluorescence intensity inside ROI **(A)**. The green signal increase informs about F-actin nucleation linked to the comet development by stimulus coupled to the red fluorescence decrease explained by granule disappearing inside ROI by actin propelling. The arrow indicates the beginning of stimulation in the represented time. **(C)** XY paths for F-actin tail (green) and vesicle (red) centroids from ROI **(A)** by particles spatial tracking analysis during cell stimulation. Yellow line shows colocalizated paths between both elements during stimulus time. **(D)** Top polar plane showing other F-actin aggregate (green) and the nearest vesicle (red) inside ROI before stimulation. **(E)** XT movements squeme of F-actin comet (green) and the nearest vesicle (red) from ROI **(D)** during stimulation. **(F)** XYT movement reconstruction of the comet and the nearest vesicle from the ROI **(D)** by 3D projection during stimulation. Scale bars: represent 1 μm.

In our experiments studying EGFP-LifeAct and RFP-NPY expression, we observed one exception to the transport of organelles described above, this involving the infrequent rapid movement of granules with F-actin tails (*N* = 11 comets in 150 stimulated cells). The examples provided in Figure 8 are representative from 11 events found in 150 stimulated cells. These “comet” like structures originated where F-actin aggregates contacted vesicles laterally (see circle in Figure 8A). In this case, the F-actin involved seems to be recruited as soon as the stimulus initiated (“*de novo*” F-actin: Figure 8B), while the granule seems to be the site of initial recruitment. The granules affected were transported at a speed of 0.1 μm/s, 3 times faster than those associated with local changes (see Movie 8), and they moved toward the plasma membrane (Figure 8C). During this movement the vesicle and its F-actin tail displayed lateral contacts that were evident in X/Y images (Figure 8D and Movie 9). These contacts generated an oscillatory lateral movement (Figure 8E) that finally propelled the vesicle in a helicoid manner (Figure 8F).

## Discussion

### F-actin dynamics during stimulation involve subtle local changes rather than general cortical transformations

The main idea behind this study was to examine in detail the changes to F-actin during cell stimulation, considering that they are likely to be local and subtle rather than general and of considerable magnitude. In effect, local expansion and retraction produces displacements of 0.5–0.6 μm that have a relatively low or no impact on the overall size of the cells, which are around 20 μm in diameter. This is consistent with earlier evidence of the local nature of F-actin fragmentation (Perrin and Aunis, 1985; Trifaro and Vitale, 1993; Vitale et al., 1995) or remodeling (Giner et al., 2005; Papadopulos et al., 2015) in cultured chromaffin cells. Indeed, the detailed study of the changes in the cell perimeter and in the cortical area detected no significant variation in cell size. This may reflect compensatory changes in response to F-actin cortical expansion and retraction in the same cell. In contrast to our neuroendocrine model, significant changes in the size of cells following stimulation have been reported in other secretory systems, such as in mast cells and beta-cells (Semino et al., 1990; Levi-Schaffer et al., 2000).

We also show here that the magnitude of these changes is temperature-dependent, reaching a remarkable displacement of 0.6 μm at 30°C, double of the size of a typical granule. Moreover, these local expansions and retractions are dependent on the activity of contractile systems, such as that involving F-actin and myosin II. As such, they would be expected to be sensitive to changes in temperature within the physiological range for exocytotic responses (Walker et al., 1996; Dinkelacker et al., 2000; Gil et al., 2001).

These changes that are transient are relevant for exocytosis because affect the areas of the plasma membrane where the secretory activity is more intense. In consequence these processes are related with the specific function of these neurosecretory cells. and could be distinguish from other cellular processes involving F-actin-myosin II activity such as membrane blebbing associated with cell motility (Fackler and Grosse, 2008).

### Organelles are transported in a coordinated fashion during F-actin re-organization

The movements observed are restricted to specific areas of the F-actin/myosin II cortical structure and they could fulfill different roles, such as the re-organization of the subcortical region to facilitate secretion in the case of expansions, or the endocytotic cycle in terms of retractions. Indeed, we demonstrate these changes are associated with the transport of vesicles and other organelles like mitochondria in a coordinated manner. In this sense, our data agrees well with the reported role of myosin II in coordinating the recruitment of vesicles during exocytosis, acting as a “casting net” that replenishes the “docking” zones that are exhausted after long lasting or repetitive cell stimulation (Papadopulos et al., 2015). Nevertheless, the data presented indicate that such a transport mechanism could be used to move entire zones of cytoplasm carrying different organelles in both directions, toward the plasma membrane in the case of expansion and recovering organelles from the sub-plasmalemmal area by retraction.

During this coordinate transport chromaffin vesicles and mitochondria could interact actively with F-actin fibers through a direct link with motors like myosin V (Rose et al., 2003; Rudolf et al., 2003) or myosin VI, as recently described (Tomatis et al., 2013). This idea is clearly supported by the movement of vesicles in association to F-actin fibers in TIRFM experiments (Lang et al., 2000).

Alternatively, organelles can move in an F-actin-dependent manner when they are found in the interior of cytoskeletal “cages,” structures that are dynamic in nature. The presence of these F-actin cages and their movement in association with the activity of myosin II has been demonstrated in both the cortical area (Giner et al., 2007), and in the cell interior (Neco et al., 2003; Giner et al., 2005). The fact that we observed the movement of entire zones of the cytosol encompassing vesicles and mitochondria supports this notion. Furthermore, the careful analysis of the size of F-actin limited cavities also indicates significant changes during cell stimulation.

### Other minor transport systems cooperate with F-actin displacement, such as F-actin comets

The majority of organelles that move actively during secretion is clearly associated with the movement of pre-existing F-actin fibers and cages, apparently coordinated within the global displacement of these structures. Nevertheless, we found that vesicles can also be directly propelled by F-actin tail comets that emerge from the F-actin aggregates at the membrane of chromaffin granules during stimulation, although this form of transport occurs less frequently. The formation of these propulsive F-actin comet tails was first described in bacteria in relation to the movement of pathogenic *Listeria* through the cytoplasm of mammalian host cells (Loisel et al., 1999). Subsequently, these comets were shown to propel endosomes and lysosomes in *Xenopus* eggs (Taunton et al., 2000). As with chromaffin granules, the F-actin was nucleated at the external membrane of the endosomes and lysosomes in *Xenopus*, a process shown to be preceded by recruitment of the N-WASP protein. Indeed, as this protein mediates the formation of “*de novo*” F-actin at the interface between the plasmalemma and chromaffin granule membrane (Gasman et al., 2004), it is reasonable to think that it might also mediate the initiation of propulsion tails in the interior of the chromaffin cell cytoplasm. At present, there is much evidence that F-actin comet tails propel endocytic vesicles in a range of systems (Collins et al., 2011), although this is the first report to our knowledge of the formation of propulsive F-actin tails during exocytosis and in the direction of the plasma membrane.

### An integrated model of F-actin mediated organelle transport during secretion in chromaffin cells

**Figure 9:**
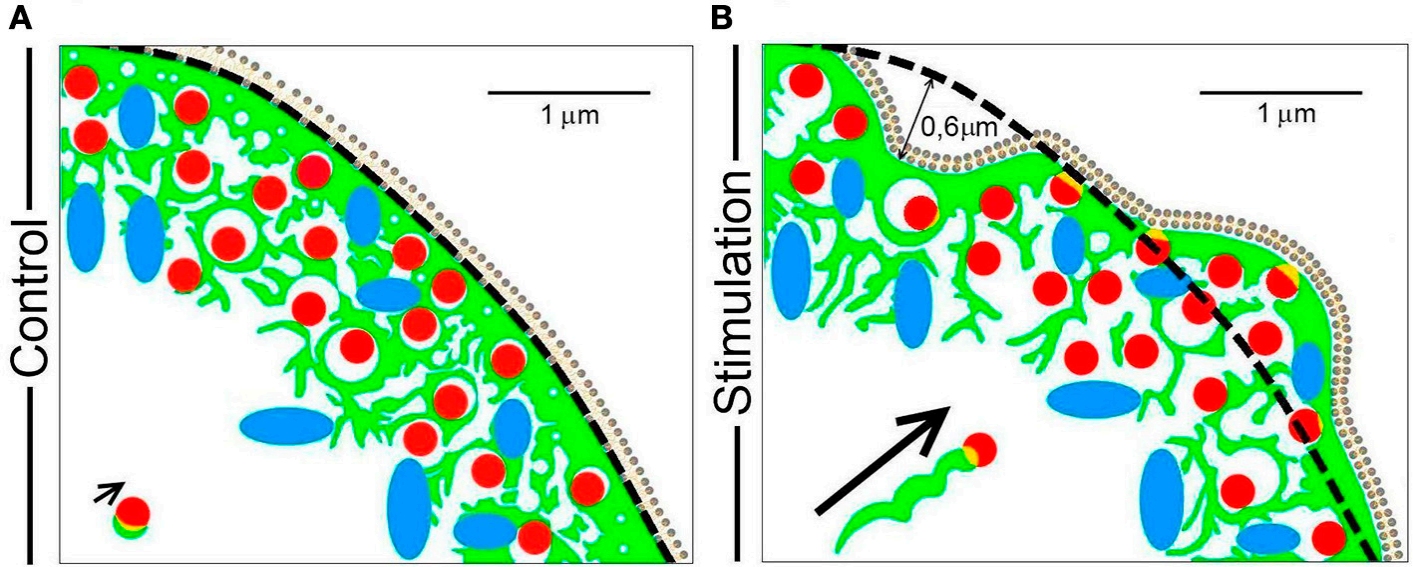
A model of the mechanisms by which F-actin drives organelle movement during chromaffin cell secretion. The different pleiotropic mechanisms utilized by F-actin (green) to transport chromaffin vesicles (red) and mitochondria (blue) during cell stimulation are summarized in the scheme. **(A)** The resting F-actin cortex in which organelles are embedded has a dense sub-membrane layer (first 0.3–0.5 μm) and a multicage zone (extending 1–1.5 μm). **(B)** During stimulation, regions of the cell periphery expand and retract, which drives the movement of the embedded organelles over a distance of about 0.5–0.6 μm. At the same time, the cage area increases leaving more space for organelle movement. A small population of vesicles can be transported via F-actin comet tails (see the arrow indicating this type of displacement). Scale bars: represent 1 μm.

The data presented here emphasizes that different mechanisms are utilized by F-actin to drive organelle transport during the secretory process in a neuroendocrine model such as chromaffin cells in culture. Therefore, we have devised a simplified scheme that models these different events (Figure 9), depicting the initial state of the cortical cytoskeleton and its associated granules and mitochondria at rest (Figure 9A), as well as the changes driven by F-actin in response to stimulation (Figure 9B). In the resting state, F-actin is organized as a dense network of cross-linked fibers beneath the plasma membrane (green structures in Figure 9A), impeding access of the majority of vesicles (red) and mitochondria (blue) to release sites. This dense barrier is wider than the size of 1–2 vesicles (c.a. 0.3–0.5 μm) and it is continuous with a complex system of F-actin cages to which organelles are either attached or embedded, at a distance of around 1–1.5 μm from the cell cortex.

During cell stimulation, the F-actin cortex experiences local changes in the form of expansions and retractions, displacing the F-actin cytoskeleton and its associated organelles by a distance of 0.5–0.6 μm (Figure 9, panel B). In addition to this movement, the size of the empty spaces left by the cytoskeletal cages is also altered, increasing the space in which organelles can move (the spaces limited by green structures in panel B of Figure 9). Therefore, the movement of the majority of vesicles and organelles is a combination of that driven by their direct association with F-actin structures and the access they have to space left by the changes in the same F-actin network.

In addition to these dominant mechanisms, F-actin also promotes the direct movement of a small proportion of vesicles during stimulation via tail comets, such as some vesicles associated with F-actin aggregates (see short arrow in Figure 9A). During stimulation, these vesicles move in a helical manner toward the cell membrane, propelled by the polymerization of F-actin in the form of a comet that emerges from the F-actin associated with the granule membrane (long arrow in Figure 9B).

Together, these mechanisms used by F-actin ensure the movement not only of vesicles but also, that of relevant organelles, such as mitochondria. In this way the replenishment of the granules exocytosed can be accomplished and the requirements of the secretory process for calcium and other elements fulfilled. These transient changes are clearly different from the continuous transport of organelles that serve to locate mitochondria and granules in different subpopulations (Gimenez-Molina et al., 2017). From the physiological point of view the transport of mitochondria to the immediate vicinity of secretory sites may be relevant for different reasons; for example it may increase the rate of calcium sequestration controlled by the presence of both mitochondria and the ER (Garcia-Sancho, 2013), especially if this mitochondrial transport is enhanced by repetitive stimulations and therefore calcium elevations are particularly large. Since mitochondrial presence may control calcium levels in the proximity of the secretory machinery it may be also influencing the mode of vesicle fusion (full fusion vs. kiss and run) under strict calcium regulation (Ales et al., 1999).

In addition to trafficking, F-actin-myosin II plays a fundamental role in the fusion mechanism itself (Neco et al., 2004) and this implies the translocation of vesicles in close apposition with the membrane, this type of displacement could be studied in a near future with the spatial resolution of the new superesolution microscopy techniques as demonstrated in other neurosecretory systems (Izeddin et al., 2011; Urban et al., 2011; O'Loughlin et al., 2018).

## Author contributions

YG, JV, and MF, performed and analyzed the experiments. YG, JV, SV, and LG designed the experiments. YG and LG wrote, reviewed, and edited the manuscript. All authors approved the final manuscript.

### Conflict of interest statement

The authors declare that the research was conducted in the absence of any commercial or financial relationships that could be construed as a potential conflict of interest.
