# Supplementary figures and images for: Multiple Mechanisms Driving F-actin-Dependent Transport of Organelles to and From Secretory Sites in Bovine Chromaffin Cells
Source: Front Cell Neurosci. 2018 Oct 9;12:344. doi: 10.3389/fncel.2018.00344 (PMC6190647; doi:10.3389/fncel.2018.00344)

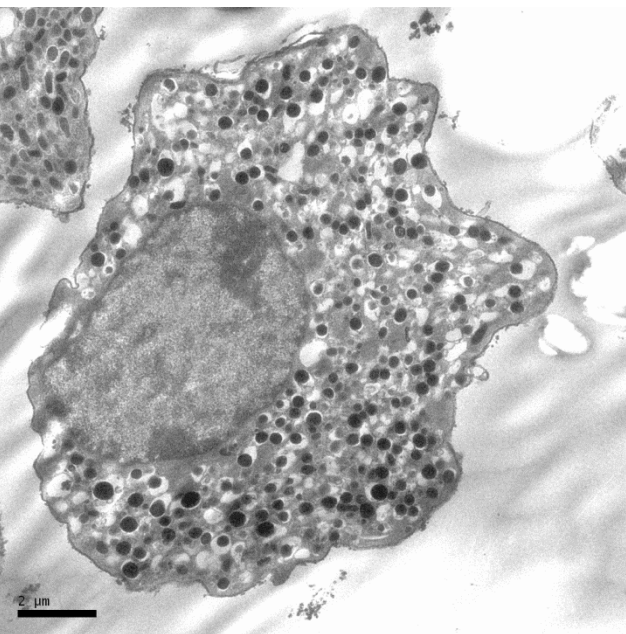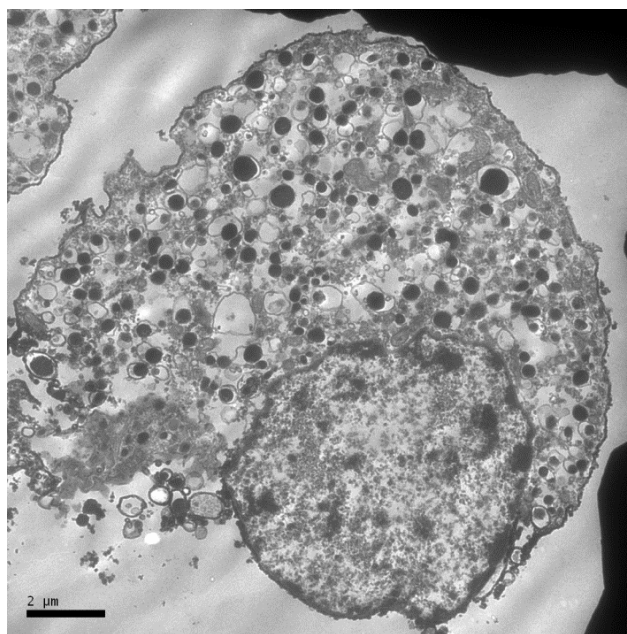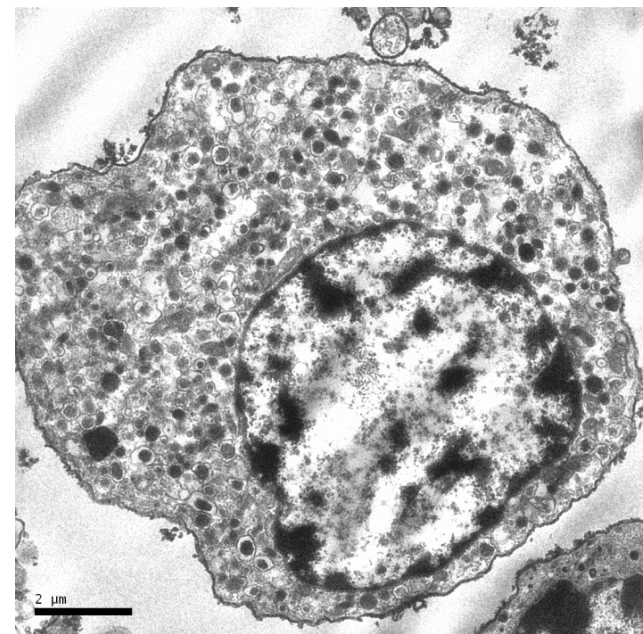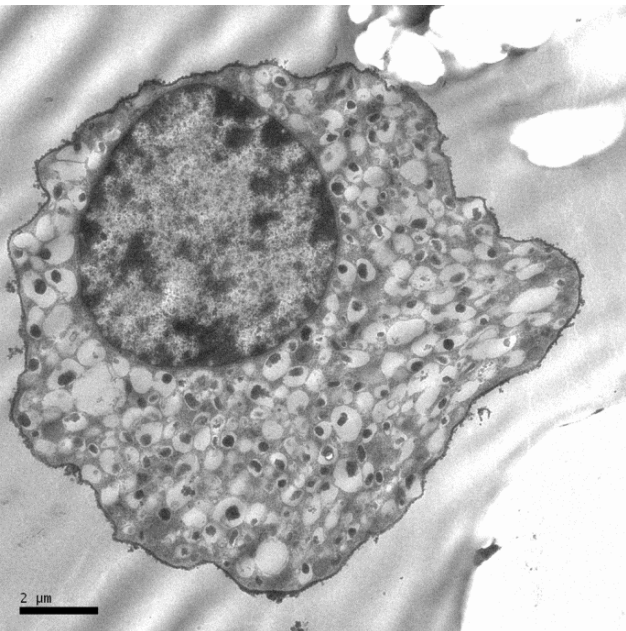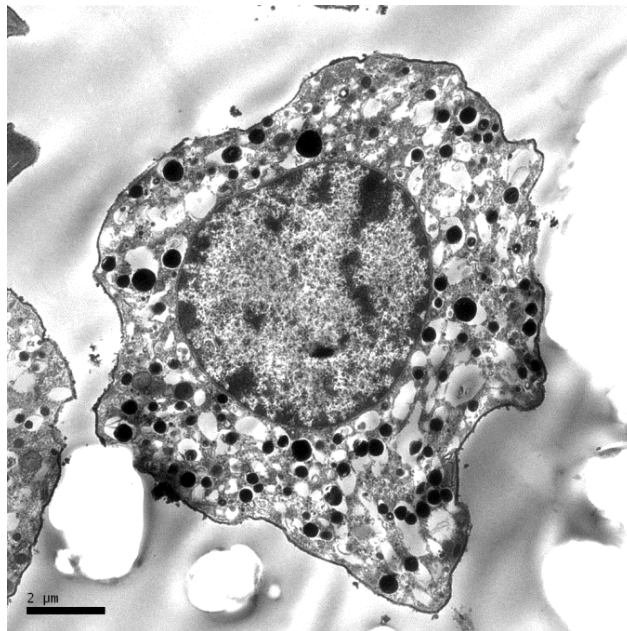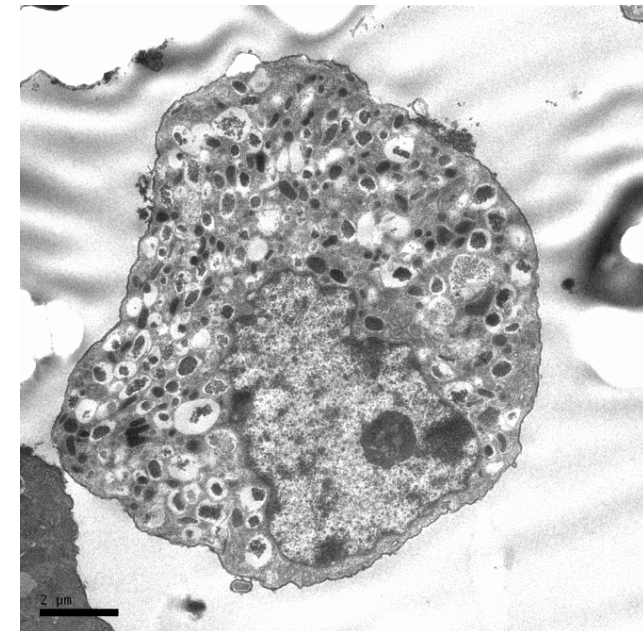

Supplement: Supplementary file 10 [file Data_Sheet_1.PDF]
