# Supplementary material for: Multiple Mechanisms Driving F-actin-Dependent Transport of Organelles to and From Secretory Sites in Bovine Chromaffin Cells
Source: Front Cell Neurosci. 2018 Oct 9;12:344. doi: 10.3389/fncel.2018.00344 (PMC6190647; doi:10.3389/fncel.2018.00344)
Supplement: Supplementary file 11 [file Image_1.pdf]

ADDITIONAL FIGURES

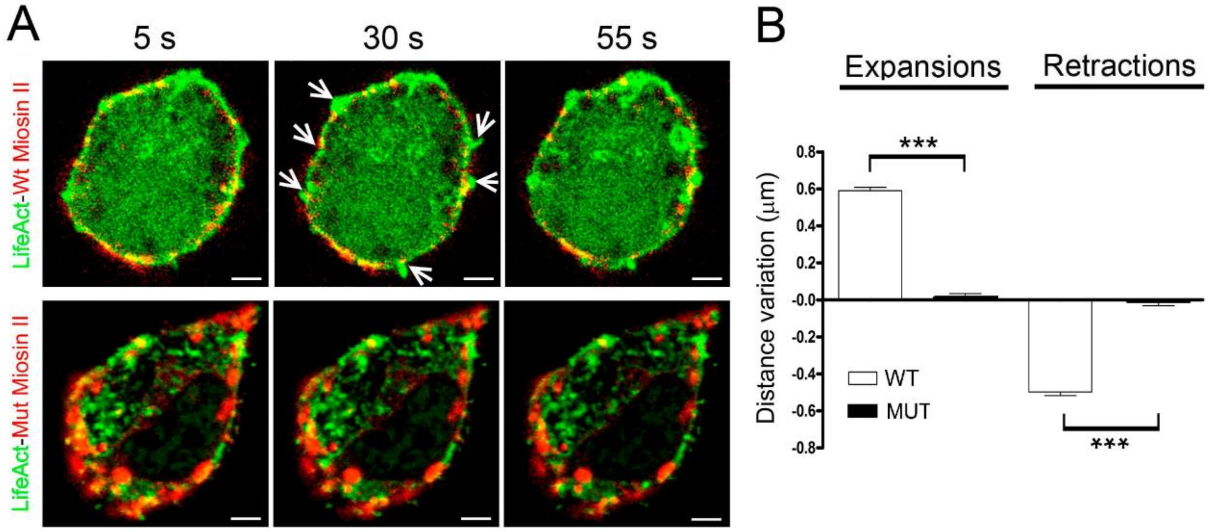

Additional Figure 1. Myosin II controls local F-actin expansion and retraction events. (A) Confocal fluorescence images of representative stimulated chromaffin cells by KCl depolarization expressing EGFP-LifeAct (green) combined with wild type and mutant version of RFP-Miosin II (red) registered at 30ºC for 5 s (before stimulation), 30 s (during stimulation) and 55 s (after stimulation). Arrows indicate local changes location. (B) Mean ± SEM values of the normalized variation in distance of the F-actin barrier at 30 s for expansion and retraction events (n=10 cells; n=30 zones). Statistical significance was assessed by Two way ANOVA: *** P<0.001. Scale bars: 1 µm.

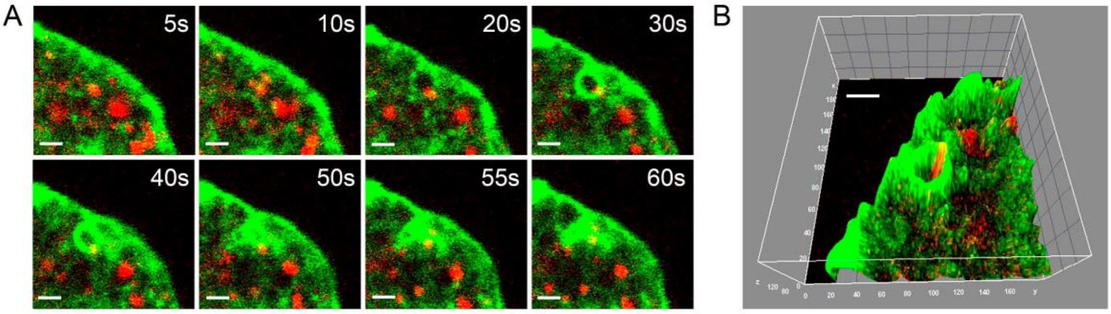

Additional Figure 2. Temporal evolution of the formation of peripheral F-actin rings during stimulation. (A) Confocal fluorescence images of a cortical section in the equatorial plane from a stimulated chromaffin cell expressing EGFP-LifeAct (green) and RFP-NPY (red) showing the temporal evolution of a F-actin ring (green) and the attached vesicle (red). The numbers indicate the acquisition time. (B) Dual channel XYZ projection of the section represented at 30s. Scale bars: 1 μm.

MOVIE DESCRIPTIONS

Movie 1. Cortical F-actin cytoskeleton local changes from a stimulated chromaffin cell at 22ºC. Movie of a time series of confocal fluorescence images from the equatorial plane of a representative stimulated bovine chromaffin cell expressing EGFP-LifeAct. This movie shows local changes in F-actin profile (arrows indicates changes location) under stimulation by KCl depolarization ("S" indicates stimulus time). This movie is related to Fig 1 and the time scale is 5 times accelerated.

Movie 2. Cortical F-actin cytoskeleton local changes from stimulated chromaffin cell at 30ºC. Confocal fluorescence movie in an equatorial plane of a representative stimulated bovine chromaffin cell expressing EGFP-LifeAct. This video shows local changes in F-actin profile (arrows indicates changes location) with an increased magnitude comparing movie1 at 22 ºC demonstrating the temperature influence on local changes under stimulation by KCl depolarization ("S" indicates stimulus time).This movie is related to Fig 2 and 5x accelerated.

Movie 3. F-actin cytoskeleton local changes from stimulated chromaffin cell under over expression of wt Miosin II. Confocal fluorescence movie in an equatorial plane of a representative stimulated bovine chromaffin cell expressing EGFP-LifeAct (green) and DsRed-wt-RLC (red). This video shows local changes in F-actin profile demonstrating that Miosin II acts as a molecular motor for F-actin dynamics allowing local changes under stimulation by KCl depolarization ("S" indicates stimulus time).This movie is related to Fig 2 and 5x accelerated.

Movie 4. Local changes abolition in cell profile from a stimulated chromaffin cell under over expression of Mutant Miosin II. Confocal fluorescence movie in an equatorial plane of a representative stimulated bovine chromaffin cell expressing EGFP-LifeAct (green) an unphosphorylatable DsRed-T18A S19A-RLC (red). This video shows the lost of F-actin fibber dynamics and consequently the abolition of local changes demonstrating Miosin II role in governing local changes development under stimulation by KCl depolarization ("S" indicates stimulus time).This movie is related to Fig 2 and 5x accelerated.

Movie 5. F-actin cytoskeletal cavities remodeling during stimulation. Confocal fluorescence movie of a representative cortical section in equatorial plane from a stimulated bovine chromaffin cell by KCl depolarization ("S" indicates stimulus time) at 30ºC expressing EGFP-LifeAct in order to visualize changes in the size and number of the F-actin cytoskeletal cavities. This movie is related to Fig4 and 5x accelerated.

Movie 6. F-actin cytoskeleton rings. Confocal fluorescence movie of a representative cortical section in equatorial plane from a stimulated bovine chromaffin cell by KCl depolarization ("S" indicates stimulus time) at 30ºC expressing EGFP-LifeAct and RFP-NPY in order to observe rings temporal evolution and vesicular linking. This movie is related to Additional Figure 1 and accelerated 5x.

Movie 7. Vesicular transport by F-actin cortical barrier retractions and expansions. Confocal fluorescence movie of a representative cortical section in equatorial plane from a stimulated bovine chromaffin cell by KCl depolarization ("S" indicates stimulus time) at 30ºC expressing EGFP-LifeAct and RFP-NPY in order to show vesicles displacement driven by F-actin fibers retractions and expansions.. This movie is related to Fig5 and accelerated 5x.

Movie 8. F-actin comet development. Confocal fluorescence movie of a representative cortical section in equatorial plane from a stimulated bovine chromaffin cell by KCl depolarization ("S" indicates stimulus time) at 30ºC expressing EGFP-LifeAct and RFP-NPY in order to visualize the F-actin comet evolution by F-actin polymerization and the consequent vesicular propulsion effect. This movie is related to Fig7 (A-C).

Movie 9. F-actin comet´s trajectories. Confocal fluorescence movie at top polar plane of a representative stimulated bovine chromaffin cell by KCl depolarization ("S" indicates stimulus time) at 30ºC expressing EGFP-LifeAct and RFP-NPY in order to show the comets helicoid movement and linked vesicular helicoid displacement. This movie is related to Fig 7 (D-F).
